# ﻿Tadpole soup: Chinantec caldo de piedra and behavior of *Duellmanohylaignicolor* larvae (Amphibia, Anura, Hylidae)

**DOI:** 10.3897/zookeys.1097.76426

**Published:** 2022-04-21

**Authors:** Carlos A. Flores, Medardo Arreortúa, Edna González-Bernal

**Affiliations:** 1 CIIDIR Unidad Oaxaca, Instituto Politécnico Nacional, Laboratorio de Ecología de Anfibios (ECA), Hornos 1003, Col. Noche Buena, 71230, Santa Cruz Xoxocotlán, Oaxaca, Mexico CIIDIR Unidad Oaxaca, Instituto Politécnico Nacional Santa Cruz Xoxocotlán Mexico; 2 CONACYT - CIIDIR Unidad Oaxaca, Instituto Politécnico Nacional, Laboratorio de Ecología de Anfibios (ECA). Hornos 1003, Col. Noche Buena, 71230, Santa Cruz Xoxocotlán, Oaxaca, Mexico CONACYT - CIIDIR Unidad Oaxaca, Instituto Politécnico Nacional Santa Cruz Xoxocotlán Mexico

**Keywords:** Amphibian, consumption, hot-rock cookery, Mexico, natural history, stream dwellers

## Abstract

Although amphibian consumption by humans has been reported globally, this practice is not well studied despite its direct implications to the decline of amphibian populations. The International Union for Conservation of Nature (IUCN) recognizes the need to document the use and trade of species to be considered in assessing their extinction risk. Here the consumption of *Duellmanohylaignicolor* tadpoles is documented. It is a micro endemic species categorized as Near Threatened (NT) consumed in a traditional dish called “caldo de piedra” (stone soup) prepared by the Chinantec people (Tsa Ju Jmí’) in Oaxaca, Mexico. Through conversations with local people and stream monitoring, the behavior of tadpoles of this species was documented and aspects of their exploitation and habitat use described. Places where caldo de piedra is still consumed were determined and using a spatial analysis with Geographic Information Systems, the distribution of the species in relation to those localities was analyzed. A number of other areas where tadpoles of this species might also occur and be exploited is predicted. In conclusion, the school behaviour, surface feeding, and the preference for deeper waterbodies that these tadpoles exhibit makes them vulnerable to being caught in large quantities. As they are consumed locally, are not commercialized, and the species distribution range is wider than caldo de piedra consumption, this implies a low risk for their populations. However, the tadpoles’ reliance on streams with depths x̄ = 60 cm and flux x̄ = 0.65 m/s reduces the availability of sites for their optimal development.

## ﻿Introduction

Amphibians are a vertebrate group at high extinction risk due to habitat transformation, alien species introduction, contamination, emergent diseases, climate change, and human exploitation (Kats and Ferrer 2003; Whittaker et al. 2013). Currently, at least 41% of all known anuran species are at risk and almost half show population declines (Hoffmann et al. 2010; Stuart et al. 2010; IUCN 2021a). Of the various ways that humans use amphibians, their use as food is poorly studied (Tyler et al. 2007; Gratwicke et al. 2010; Akinyemi and Ogaga 2015; Grano 2020). Amphibian consumption by humans has received far less attention than mammal and bird consumption (Ibarra et al. 2011; Chaves et al. 2019; Dobson et al. 2019; Ripple et al. 2019) and it is usually reported by social scientists who frequently fail to identify the species involved. Amphibian consumption data are often present in the grey literature and not evaluated in the context of species risk (González-Hernández 2019). Frogs have been part of the human diet since the early Pleistocene and archeological remains suggest that this consumption was not random (Kyselý 2008; Blasco et al. 2011). This practice continues in different cultures across the globe (Cooke 1989; Onadeko et al. 2011; Akinyemi and Ogaga 2015; Ohler and Nicolas 2017) and in some cases it can become a tangible species threat. For example, in Indonesia, *Fejervaryacancrivora* (Gravenhorst, 1829) (Least Concern) and *Limnonectesmacrodon* (Duméril & Bibron, 1841) (Least Concern), are exploited for both local consumption and large scale exportation. Since these species are not raised in captivity, it can be assumed that all consumed individuals are collected from the wild (Kusrini and Alford 2006).

In general, amphibian consumption studies focus mainly on adult anurans with less attention paid to tadpole exploitation, even though larvae consumption might imply the need to capture larger numbers of individuals to match an equivalent nutritional yield to that of adult consumption. For example, tadpole consumption of the Western Ghats (India) endemic frog *Nasikabatrachussahyadrensis* (Biju & Bossuyt, 2003; Purple frog), reaches up to 50% of the available individuals yearly and the proportion of collected/available tadpoles increased to 70% from 2008 to 2012, representing a direct threat to the species’ survival (Thomas and Biju 2015).

Despite a genuine concern about amphibian overexploitation for human consumption and its effects on species population sizes (Stuart et al. 2004; Kusrini and Alford 2006; Onadeko et al. 2011; Talukdar and Sengupta 2020), data on species use are still scarce in relation to other threats considered by the IUCN. For example, although amphibian cultural importance and use has been widely documented in México, for Oaxaca, the state with the richest amphibian diversity in the country, only 7.55% (12/159) of the amphibian species present a general scheme of use according to the IUCN (Parra-Olea et al. 2020; Mata-Silva et al. 2021; IUCN 2021b).

Regarding tadpole consumption by humans, it is only superficially mentioned in historical writings with the species remaining unidentified (Aguilar and Luría 2016). Even now, in rural or indigenous communities where tadpoles are eaten, researchers tend to mention it only anecdotally with no formal studies on this practice.

During herpetofauna surveys in Santa Cruz Tepetotutla, our field guide Pedro Osorio-Hernández brought to our attention the local consumption of tadpoles in a soup called “caldo de piedra” (stone soup in English). Caldo de piedra is an ancient dish that is traditionally prepared on the bank of the river, using a container or rock holes in which river water, fish meat, vegetables, and raw seasonings are placed and cooked by adding heated stones. In order to study this activity, identify the tadpole species involved, and document other biological and cultural information, we conducted conversational interviews, rivers and streams surveys, spatial analysis through Geographic Information Systems, and a literature review. Here we report that tadpoles of the Sierra Juarez brook frog *Duellmanohylaignicolor* (Duellman, 1961) are consumed in caldo de piedra in the Chinantla region, in Oaxaca Mexico. We also report on aspects of the tadpole’s behavior and natural history relevant to its exploitation and survival.

## ﻿Materials and methods

### ﻿Study area

**Figure 1.**
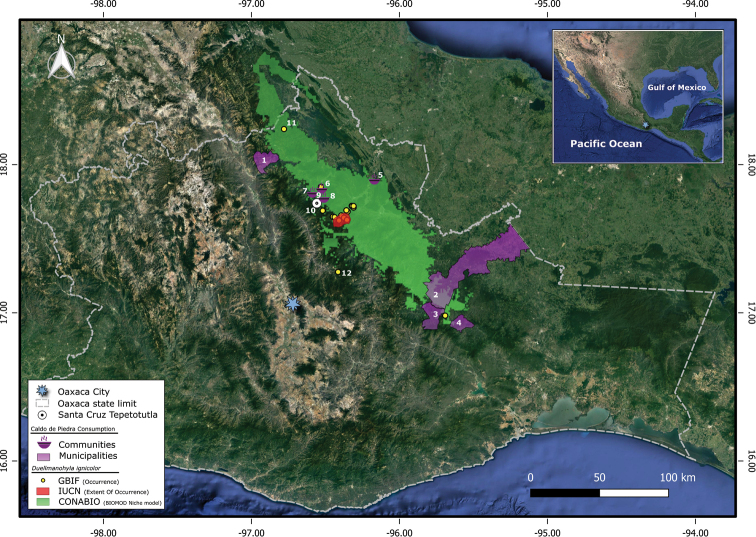
Study site. Santa Cruz Tepetotutla locality in Oaxaca, Mexico represented by a white dot. The red polygon represents the Extent Of Occurrence elaborated by IUCN. The green polygon represents the CONABIO model. GBIF occurrences are represented by yellow dots. Municipalities and communities where caldo de piedra is consumed are represented by areas in purple and by a purple soup icon respectively.

Santa Cruz Tepetotutla (17.7391°N, -96.5582°W) is a Chinantec indigenous community located in the southwest portion of San Felipe Usila municipality in the state of Oaxaca, Mexico (Fig. 1). It forms part of an indigenous region known as “La Chinantla”, which is subdivided in three ecophysiographic subregions known as higher, medium, and lower Chinantla due to altitudinal differences (de Teresa 1999). It includes 14 municipalities and 258 communities. Throughout the region, Chinantec language is spoken in 11 variants (INEGI 2010). In their own language, Chinantecs refer to themselves as *Tsa ju jmí*’, which means “people of the ancient word” (INPI-INALI 2020).

**Figure 2.**
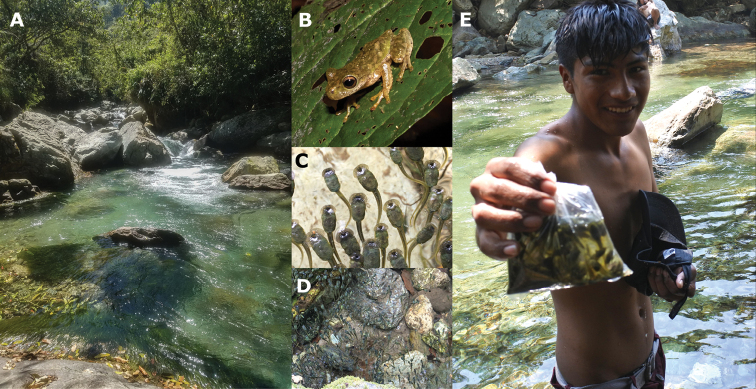
Aspects of *Duellmanohylaignicolor* natural history and use **A** characteristic pools in the Tlacuache river where tadpoles aggregate **B***D.ignicolor* adult **C***D.ignicolor* tadpoles **D** school forming behavior **E** a teenager collecting tadpoles with a cap and keeping them in a plastic bag. Photographs: (**A, C, D, E**) by Edna González-Bernal; (**B**) by Medardo Arreortúa.

Santa Cruz Tepetotutla preserves 9,670 ha of montane cloud forest under the Indigenous and Community Conserved Area (**ICCAs**) modality, certified by National Protected Area Commission in Mexico (**CONANP**). It supports the presence of several threatened species (Simón-Salvador et al. 2021). The main vegetation is montane cloud forest, with several streams filling two main waterways, the Tlacuache river (Fig. 2A) and the Perfume River, which discharge their waters into the Usila River that finally reaches the Presidente Miguel de la Madrid Hurtado dam in the lower Chinantla (INEGI 2014).

### ﻿Species

*Duellmanohylaignicolor* (Fig. 2B) is an hylid frog endemic to Oaxaca, Mexico and restricted to the Sierra Madre de Oaxaca (**SMO**) physiographic region (Duellman 1961; Duellman 2001; Ortiz- Pérez et al. 2004). According to the IUCN it is catalogued as Near Threatened (**NT**) and it has not been considered as used in trade or for human consumption (IUCN 2020).

### ﻿Interviews

We conducted interviews with men and women in the community to ask mainly if caldo de piedra was still prepared locally. If it was, we asked which tadpoles were used in its preparation, where, when, and by whom they are collected, how they taste, and how they know which are edible or not.

### ﻿River surveys

As the interviewees referred to tadpoles captured at the main river (Río Tlacuache); we surveyed it at accessible areas along five sections of approximately 50 m long, looking for tadpoles with the described behavior. Once found we corroborated that those were the tadpoles used to prepare caldo de piedra by talking to a family that was eating caldo de piedra at that moment by the river. Species identification was made according to literature (Duellman 1961).

We conducted stream surveys to determine tadpole presence and stream characteristics in other waterways in the region. A total of ten waterways in addition to Río Tlacuache was surveyed in 50 m long sections. The characteristics assessed were depth, width, and water current speed measured with a flow meter Flow Watch 30 (JDC Electronics SA). Surveys took place between April and August in 2019.

### ﻿Literature review and spatial analysis

To give a better perspective on caldo de piedra consumption and explore potential areas where it can be prepared with *D.ignicolor* tadpoles (Fig. 2C) as an ingredient, we reviewed ethnographic research conducted in the region related to this cooking style and cuisine. An approximation of the likelihood of *D.ignicolor* tadpoles being a component of contemporary caldo de piedra, was made with a spatial analysis that compared the species distribution with areas where this dish is consumed. Available geospatial information for *D.ignicolor* was obtained. These included the potential distribution area elaborated by the Comisión Nacional para el Conocimiento de la Biodiversidad (**CONABIO**) (Ochoa-Ochoa and Flores-Villela 2016), the Extent Of Occurrence (**EOO**) polygon elaborated by IUCN, and the species occurrence records from Global Biodiversity Information Facility (GBIF 2021). The data was compared with the sites where the elaboration of the caldo de piedra is reported in literature. The information was analyzed on QGIS 3.16.4 (Quantum GIS Development Team 2021).

## ﻿Results

### ﻿Interviews

Caldo de piedra with *Duellmanohylaignicolor* tadpoles as an ingredient is still prepared and eaten in Santa Cruz Tepetotutla, although these days the main animal protein in the soup is farmed *mojarra* (*Oreochromisniloticus* (Linnaeus, 1758) or *Coptodonrendalli* (Boulenger, 1897)). During April when tadpoles are abundant, people prepare the soup both at the river and at home. Children are usually the ones that capture the *D.ignicolor* tadpoles, especially during the Easter holidays (April) when they go swimming in the river. The tadpoles are captured in plastic bags, by hand, and even with hats or caps. Innards are removed by squeezing the tadpole's body with the fingers. Once “cleaned”, they are placed in a bowl with tomato, onion, chili, salt, wild coriander, and water and then the mixture is brought to a boil by adding small hot stones until the soup is cooked.

*Duellmanohylaignicolor* tadpoles are considered cleaner and thus more edible than tadpoles from other stream species because they swim at the water surface (Fig. 2D). The tadpoles of other species in the streams are bottom dwellers and are perceived of as dirty because of their contact with the sediment. The tadpoles that are consumed are between Gosner stages 30 and 35. Later stage larvae with easily visible legs are not considered edible. Interviewees described the tadpoles as having a delicious fish-like flavor (Fig. 2E).

### ﻿*Duellmanohylaignicolor* tadpole habitat use and behavior

Of the ten surveyed streams in the locality, we found *D.ignicolor* tadpoles in only one of them (Bado stream) apart from the main river (Río Tlacuache). *Duellmanohylaignicolor* tadpoles prefer deeper pools available at the edge of the stream (x̄ = 60 cm ± SD = 7.6, *n* = 12 in used streams vs. x̄ = 11.9 cm ± SD = 6, *n* = 54 in unused streams). Water bodies with faster currents (x̄ = 0.65 m/s ± SD = 0.11, *n* = 12 in used streams vs. x̄ = 0.31 m/s ± SD = 0.26, *n* = 54 in unused streams) and that are deeper at the center (x̄ = 81.2 cm ± SD = 6.32, *n* = 12 in used streams vs. x̄ = 20.8 cm, ± SD = 12.90, *n* = 54 in unused streams). Values of utilized waterways include measurements taken at the main river. Kruskal-Wallis test conducted on all measurements showed statistical differences *P*<.0001.

*Duellmanohylaignicolor* tadpoles can be found at the river edge using pools formed by rocks. Most of the time, they are near to the water surface with head-up positions forming schools. It is possible to find groups composed of more than 100 individuals in ~ 4 m^2^ (Fig. 2C, D). While in this position, they continuously move their mouthparts which suggests they might be feeding on suspending particles dragged by the water current or that land on the water surface. If disturbed by any unusual movement in the water, they move towards the rocks and cling to them using their large oral disc. They can also hide in the leaf litter or beneath the rocks located at the bottom of the pool. Large oral discs in this species are considered an adaptation to living in fast-moving currents (Caldwell 1974). At the study site, individuals at Gosner stage 36 reached up to 52 mm in total length.

At Río Tlacuache in Santa Cruz Tepetotutla, this species shares microhabitat with *Ptychohylazophodes* (Campbell & Duellman, 2000) and *Inciliusvalliceps* (Wiegmann, 1833). In Arroyo Bado, they co-occur with *P.zophodes*. In contrast to *D.ignicolor* tadpoles, *P.zophodes* and *I.valliceps* tadpoles are benthic feeders so they spend most of the time at the bottom of the pools.

### ﻿Caldo de Piedra and amphibian consumption by Chinantec people (Tsa Ju Jmí’)

Caldo de Piedra was exclusively prepared by men, who dug a hole in the river sand and covered it with pozol leaves (*Calathealutea* (Aubl) E. Mey. ex Schult, 1822) to prevent the water from escaping (river rock holes are also used). Chili, vegetables, and salt were placed inside and with a branch, some egg-sized rocks, previously heated in a campfire, were added in order to cook the food. When the water began to boil, a fish without entrails was added and cooked for 10–15 minutes. Finally, the broth was served in a plate made with pozol leaves or in a “Jícara”, a bowl made of *Crescentiacujete* Linnaeus, 1753, and it was accompanied with “tortillas” (Weitlaner 1951; Bost 2009). There is no agreement on when and by whom caldo de piedra was invented. Its ancient origin is claimed by some inhabitants of San Felipe Usila as a “millenary cooking practice uniquely developed by fishermen in his community” (Brulotte and Starkman 2014). The dish is based on a gendered division of labor where “women bathe and wash clothes in the river, but it’s only adult men who fish and prepare the caldo. With the finished soup finally being offered to women and children” (Brulotte and Starkman 2014). Nevertheless, similar dishes are present in other Oaxacan cultures like Ayuk (Mixe) where it is named “caldo de playa” since it is made at the riverbanks (Nahmad 2003; Sánz 2015). There is evidence that this type of hot-rock cooking has been long used in by North American cultures (Thoms 2008), with the same cooking principle know to occur in Europe since the late Aurignacian (ca. 32,000–33,000 B.P.) and similar cooking technology occurring elsewhere in the world (Thoms 2009).

One of the common characteristics among the different descriptions of caldo de piedra from Oaxaca is the use of ingredients like tomato, chili, spices, salt, and fish (bobo fish *Joturuspichardi* Poey, 1860 and trout *Oncorhynchusmykiss* Walbaum, 1792) as the main base, but also river shrimp, prawns, and snails (Mejía and González 2019). However, no amphibians as either as tadpoles or adult frogs have been previously mentioned.

### ﻿Spatial analysis

There are currently two species distribution models for *D.ignicolor* (Fig. 1). The first, presented by CONABIO uses the BIOMOD platform and suggests that the distribution of the species is 6605 km^2^ including the states of Oaxaca, Puebla, and Veracruz (Ochoa-Ochoa and Flores-Villela 2016). The second model, elaborated by IUCN, uses EOO parameter and suggests that the species is restricted to only 91 km^2^ within Sierra Madre de Oaxaca physiographic region (IUCN 2021b).

Occurrence records for the species obtained from GBIF (gbibID: 1897584918, 1572339861), CONABIO and MZFC (Museo de Zoología de la Facultad de Ciencias, UNAM) (Ochoa-Ochoa and Flores-Villela 2016), include three localities outside the IUCN model (Table 1). The first corresponds to a specimen collected in Capulalpam de Méndez (Departamento de Zoología, Instituto de Biología IBUNAM, CNAR24801). The second record is an observation of several adults in a stream in San Miguel Quetzaltepec municipality (Levy N. Gray, pers. comm.). It is outside south the area of the UICN model and represents the most southern known occurrence of the species. The third record corresponds to a series of specimens collected in Ejido Clemencia, a locality in Santa María Chilchotla municipality, that represents the most northern known occurrence of the species (Ochoa-Ochoa and Flores-Villela 2016).

**Table 1.** Location of communities and municipalities referred in Fig. 1. Inclusion (✓) or exclusion (✗) in the CONABIO and UICN distribution models for *Duellmanohylaignicolor*. Species occurrence refers to real occurrence data.

Map id	Municipalities	Conabio	IUCN	Species Occurrence	Caldo De Piedra Consumption	Coordinates
1	Mazatlán Villa de Flores	✗	✗	✗	✓	18.032542°N, -96.915527°W
2	San Juan Cotzocón	✓	✗	✗	✓	17.160736°N, -95.783228°W
3	San Miguel Quetzaltepec	✓	✗	✓	✓	17.018643°N, -95.830581°W
4	Santiago Ixcuintepec	✓	✗	✗	✓	16.934397°N, -95.623581°W
	**Communities**					
5	San José Chiltepec	✗	✗	✗	✓	17.948046°N, -96.169111°W
6	San Felipe Usila	✓	✗	✗	✓	17.887505°N, -96.524692°W
7	San Juan Bautista Tlacoatzintepec	✓	✗	✗	✓	17.859707°N, -96.586562°W
8	Santiago Tlatepusco	✓	✗	✗	✓	17.825197°N, -96.509955°W
9	San Antonio del Barrio	✗	✗	✗	✓	17.758098°N, -96.556130°W
10	Santa Cruz Tepetotutla	✗	✗	✓	✓	17.739398°N, -96.558096°W
11	Ejido Clemencia	✓	✗	✓	✗	18.240000°N, -96.780000°W
12	Capulalpam de Méndez	✓	✗	✓	✗	17.275553°N, -96.414522°W

From our spatial analysis we determined that caldo de piedra is consumed in ten localities among three regions: La Chinantla (six localities), the Mixe region (three localities), and the Cañada region (one locality). From these ten localities, only six overlap with the CONABIO distribution model of *D.ignicolor* (Fig. 1), and none of them overlap with the IUCN distribution model. In only two localities (Santa Cruz Tepetotutla and San Miguel Quetzaltepec) the species occurrence has been confirmed; only in Santa Cruz Tepetotutla has it been confirmed that caldo de piedra is prepared with *D.ignicolor* tadpoles (Table 1).

## ﻿Discussion

Documenting the human use of a species is fundamental to developing conservation measures. One cause of species’ declines is human consumption linked to poor regulation. Educational programs deficient in environmental and ecological foci and few or no economic alternatives for people consuming the taxa are contributing to species’ declines. Seventeen native amphibian species out of 411 in Mexico are known to be consumed by humans: *Agalychnisdacnicolor* (Cope, 1864), *Ambystomadumerilii* (Dugès, 1870), *A.mexicanum* (Shaw & Nodder, 1798), *A.taylori* (Brandon, Maruska, & Rumph, 1981), *A.velasci* (Dugès, 1888), *A.altamirani* (Dugès, 1895), *A.granulosum* (Taylor, 1944), *A.lermaense* (Taylor, 1940), *Charadrahylataeniopus* (Günther, 1901), *Dryophyteseximius* (Baird, 1854), *Lithobatesforreri* (Boulenger, 1883), *L.tlaloci* (Hillis & Frost, 1985), *L.sierramadrensis* (Taylor, 1939), *L.montezumae* (Baird, 1854), *L.spectabilis* (Hillis & Frost, 1985), *Rheohylamiotympanum* (Cope, 1863) and *Rhinellahorribilis* (Wiegmann, 1833) (Huacuz 2002; Casas-Andreu et al. 2004; Carpenter et al. 2007; Altherr et al. 2011; Velarde 2012; Aguilar and Luría 2016; González-Hernández 2019; IUCN 2021a). All these species are consumed at their adult stage. Tadpole consumption has only been formally reported for one native species: *Lithobatesmontezumae* (Baird, 1854) and one introduced species *L.catesbeianus* (Shaw, 1802) (Casas-Andreu et al. 2004; González-Hernández 2019).

We report the first record of *Duellmanohylaignicolor* tadpole consumption in the country in a traditional soup called caldo de piedra. In Mexico, this meal is consumed in different localities in Oaxaca, but in Santa Cruz Tepetotutla, it is prepared with *D.ignicolor* tadpoles. This tadpole soup is consumed during the hottest months (April and May: Fernández et al 2012), when people go swimming in the river. Therefore, human predation of this species takes place at the main river of the locality. The rest of the year, the soup is prepared with fish.

Biological characteristics can make tadpoles of some species more exploitable than others, for example, *Nasikabatrachussahyadrensis* (Biju & Bossuyt, 2003) tadpoles are collected in large numbers due to their practice of attaching themselves in groups to rock surfaces in waterfalls. Consequently, people easily sweep large numbers off the rocks using branches (Thomas and Biju 2015). Concerning *D.ignicolor* tadpoles, we registered three aspects that contribute to their exploitation by humans: their feeding behavior, their school formation (McDiarmid and Altig 1999), and their preference for deeper water pools.

*Duellmanohylaignicolor* tadpoles feed on suspended particles by swimming near the water surface with head-up postures. While feeding, they form schools that facilitate their catch in big numbers. This schooling behavior is associated with protection against predators, temperature, and the effectiveness of feeding strategies (Blaustein and Waldman 1992; Blaustein and Walls 1995; Spieler 2003; Hase and Kutsukake 2019). However, this behavior makes them more vulnerable to human exploitation. As they swim near the water surface, people perceive them as clean and prefer them over co-occurring tadpoles that exhibit a benthic behavior. Tadpoles with benthic behavior (*P.zophodes* and *I.valliceps*) are considered dirty due to their association with sediments.

Contrary to previous studies that mention that *D.ignicolor* tadpoles use shallow gravel-bottomed pools in streams (Duellman 2001), we found them in deeper water bodies with faster currents. The preference for deeper water has been associated with an anti-predatory strategy and as a way to reduce the chances of pool drying during the dry season (Borges-Júnior and Rocha 2013). However, this strategy might increase the chances of human exploitation, as humans use deeper water bodies to swim.

Concerning habitat use, *D.ignicolor* tadpoles can be found in waterbodies with faster currents. The existence of another stream used by *D.ignicolor* tadpoles from where they are not extracted ensures their local presence. However, most of the available streams in the locality present lower currents, and are not used by tadpoles of this species, even when we observed adult individuals on those streams. Differences in microhabitat preference among developmental stages have been reported in other amphibian species but their causes remain to be studied (Afonso and Eterovick 2007; Eterovick et al. 2010).

It will be necessary to determine the impact of reduced availability of optimal streams combined with human extraction. In this sense, everyone we spoke with referred to tadpole consumption as local and without commercial purposes. Nobody reported selling tadpoles and we have not heard about any trading with them. *D.ignicolor* tadpole consumption is not as threatening as in cases where large numbers of larvae are extracted yearly, i.e., *Nasikabatrachussahyadrensis* (Thomas & Biju, 2015).

From the six communities where caldo de piedra is consumed that overlap with the CONABIO species distribution model (Ochoa-Ochoa and Flores-Villela 2016), only in one, has the species presence been confirmed. It is necessary to verify that the species occurs in the remaining five communities, and if so, whether or not it is used in tadpole soup. The new record from Santa Cruz Tepetotutla confirms that the species occurs in a locality not included in any distribution model, and it represents the only instance where *D.ignicolor* tadpoles are consumed in caldo de piedra.

The information gathered can give a better perspective of the pressure factors to which the species is exposed. The conservation status of *D.ignicolor* has been recently changed from Endangered (EN) to Near Threatened (NT) as it occurs in an Indigenous and Community Conserved Area (ICCA’s) (IUCN 2020). Even when some populations distribute within protected land, pressure factors occurring in the area like chytridiomycosis, land-use change, reduced availability of ideal streams and tadpole consumption, can affect this species.

Finally, we consider that the recent increase in studies on larval stages (Rivera-Correa et al. 2021) is of vital importance for the conservation of amphibians. A broader knowledge of biology, behavior, and the natural history of adult and larval stages (Malagoli et al. 2021) should allow the design of appropriate conservation measures for organisms with complex life cycles like amphibians.
